# Prospective study of Outcomes in Sporadic versus Hereditary breast cancer (POSH): study protocol

**DOI:** 10.1186/1471-2407-7-160

**Published:** 2007-08-15

**Authors:** Diana Eccles, Sue Gerty, Peter Simmonds, Victoria Hammond, Sarah Ennis, Douglas G Altman, the POSH steering group

**Affiliations:** 1 Somers Cancer Sciences Building Mail Point 824, Southampton University Hospitals NHS Trust, Tremona Road, Southampton SO16 6YA, UK; 2 Centre for Statistics in Medicine, Wolfson College Annexe, Linton Road, Oxford OX2 6UD, UK; 3 POSH Steering group: see after acknowledgement section for members and roles

## Abstract

**Background:**

Young women presenting with breast cancer are more likely to have a genetic predisposition to the disease than breast cancer patients in general. A genetic predisposition is known to increase the risk of new primary breast (and other) cancers. It is unclear from the literature whether genetic status should be taken into consideration when planning adjuvant treatment in a young woman presenting with a first primary breast cancer. The primary aim of the POSH study is to establish whether genetic status influences the prognosis of primary breast cancer independently of known prognostic factors.

**Methods/design:**

The study is a prospective cohort study recruiting 3,000 women aged 40 years or younger at breast cancer diagnosis; the recruiting period covers 1^st ^June 2001 to 31^st ^December 2007. Written informed consent is obtained at study entry. Family history and known epidemiological risk data are collected by questionnaire. Clinical information about diagnosis, treatment and clinical course is collected and blood is stored. Follow up data are collected annually after the first year. An additional recruitment category includes women aged 41 to 50 years who are found to be BRCA1 or BRCA2 gene carriers and were diagnosed with their first breast cancer during the study recruiting period.

**Discussion:**

Power estimates were based on 10% of the cohort carrying a BRCA1 gene mutation. Preliminary BRCA1 and BRCA2 mutation analysis in a pilot set of study participants confirms we should have 97% power to detect a difference of 10% in event rates between gene carriers and sporadic young onset cases. Most of the recruited patients (>80%) receive an anthracycline containing adjuvant chemotherapy regimen making planned analyses more straightforward.

## Background

Less than 5% of breast cancers diagnosed in the UK are diagnosed in women aged 40 years or younger although there is a rapid increase in the incidence from about 35 years of age[1].

Published retrospective studies suggest that early age at onset of breast cancer (below 35 years) is a poor prognosis factor; high grade and oestrogen receptor (ER) negative tumours appear to be more frequent in younger women[2,3]. Breast cancer arising due to a high penetrance genetic predisposition gene such as BRCA1 or BRCA2 occurs at younger average age than breast cancer in the general population and a higher proportion of young onset cases will have a genetic predisposition than breast cancer cases in general. Family history is still the most important indicator of an underlying inherited predisposition [4]. However BRCA1 and BRCA2 may account for less than 40% of all familial breast cancer; multiple low penetrance breast cancer predisposition genes are likely to account for the rest[5]. These low penetrance alleles are now beginning to be discovered through both candidate gene approaches and whole genome SNP (single nucleotide polymorphism) analyses [6-9].

Certain tumour characteristics are characteristically seen in breast cancers due to a BRCA1 gene mutation and also to a lesser degree in BRCA2 gene carriers [10-12]. For example high grade, ER, PR and HER2 negative tumours are significantly more frequent in BRCA1 gene carriers than in breast cancers in none gene carriers. In examining the effect of BRCA1 or BRCA2 status on prognosis after breast cancer diagnosis, it is important in comparing genetic with apparently sporadic breast cancer cases, to take into account known prognostically important tumour characteristics and adjuvant treatment regimens should be broadly similar in the groups being compared.

There are several areas of important clinical uncertainty regarding the management of hereditary breast cancer. It is not clear whether the prognosis for women with BRCA1 and BRCA2 related breast cancers differs compared to sporadic tumours with similar pathological prognostic indices. Current publications variously suggest that overall survival for BRCA1 gene carriers is better[13,14], the same[15,15,16] or much worse[17,18] than sporadic cancers but most published studies to date have been based on small numbers and retrospective data and are inherently unreliable.

Both BRCA1 and BRCA2 are involved in cellular DNA repair mechanisms [19]. Furthermore a number of other genes involved in DNA repair and found to be mutated at low frequencies in the general population (<5%) are currently implicated as low penetrance susceptibility alleles [6,7,20]. This, at least hypothetically and perhaps controversially, might raise some concerns about the use of mammographic screening in those at increased risk. This may also have relevance for gene carriers undergoing potentially curative treatment for early stage breast cancer since radiation therapy and chemotherapy will induce DNA damage in normal as well as malignant tissues. Furthermore preliminary evidence suggests that BRCA1 is important in the mechanism of action of the spindle poisons paclitaxel and vinorelbine. BRCA1 null tumour cells may therefore be intrinsically resistant to these agents[21].

BRCA1 and BRCA2 gene carriers are at increased risk of developing contralateral breast cancer and appear, in the longer term, to have an increased rate of new primary tumours in the affected breast (reviewed in[22]). The role and timing of prophylactic contralateral mastectomy or bilateral mastectomy in these patients remains uncertain but the possibility should be considered in this patient group.

The uncertainties surrounding the most appropriate treatment of breast cancer in BRCA1 and BRCA2 gene carriers is of critical importance and must be resolved in order that clinicians can better tailor treatment in hereditary breast cancer. There is a need for robust unbiased data to provide clear answers to the question of risk of death from the presenting cancer diagnosis versus risk of a future new primary cancer in an individual with hereditary breast cancer. Genotype and tumour phenotype should be considered so that questions of future risk reducing strategies can be given appropriate weight and timing by both patient and the treating oncologist.

We describe here the design and progress of a prospective study established to provide reliable answers to questions about the influence of genotype on tumour phenotype and prognosis.

In addition, since young onset breast cancer is uncommon, the prospective design of this very large cohort study presents many opportunities for exploring how inherited genetic traits affect tumour biology and treatment outcomes. Studies exploring how lower penetrance genes, radiological features and obesity and weight gain relate to prognosis in this cohort are some of the sub-studies currently in progress[23]. The steering group welcomes expressions of interest for collaborations. The most important criterion for selecting collaborative projects will be that the proposed research question can best be answered in a young onset, symptomatic group of patients.

## Methods/design

The Prospective study of Outcomes in Sporadic versus Hereditary breast cancer (POSH) is a large, prospective cohort study. This design was chosen to minimise ascertainment bias, facilitate identification of controls that could be matched for all potential confounding factors and ensure accurate standardised and high quality data collection.

### Aims of the study

The primary aims of the study are to determine whether:

(1) The prognosis of patients with breast cancer who harbour BRCA1 or BRCA2 gene mutations differs from non carrier patients after adjustment for age and other major prognostic indicators.

(2) Breast cancers occurring in patients with an inherited mutation in the same predisposing gene have a consistent and distinct tumour phenotype.

(3) There are differences in the pattern of distant breast cancer recurrence between patients with BRCA1 or BRCA2 gene mutations and matched non carrier patients.

#### Secondary aims

(1) To develop a validated set of pathological criteria that improve the specificity and sensitivity of methods for identifying carriers of germ-line BRCA1/2 mutations to facilitate future clinical trials based on genotype.

(2) To determine whether inherited genetic variants influence tumour biology (particularly metastatic potential)

(3) To describe the radiological features in a large cohort of young breast cancers and correlate these with genetic factors, tumour pathology and prognosis.

### Inclusion criteria

 Women diagnosed with invasive breast cancer

 Aged 40 years or younger at diagnosis

 Diagnosed between January 2000 and December 2007.

#### Plus

 Women aged 41–50 with a known BRCA1 or BRCA2 gene mutation, diagnosed with invasive breast cancer within the study period.

### Exclusion criteria

 Previous invasive malignancy (with the exception of non-melanomatous skin cancer)

 Not available for follow up

 Refuse consent to retain diagnostic and follow up data

### Ethics Approval

This study received approval from the South and West Multi-centre Research Ethics Committee (MREC 00/6/69)

### Method of recruitment

Study recruitment is supported by the National Cancer Research Network . Recruiting centres across England, Scotland, Wales and Northern Ireland. Recruitment began in April 2001. Recruitment was initially through the Southampton Breast Centre and all eligible individuals diagnosed from January 2000 were approached to participate since we could be sure that there had been no deaths of eligible women diagnosed between January 2000 and June 2001 when recruiting started in this centre. On joining the study each new centre was instructed to approach all patients as early as practical within 12 months of diagnosis, to ensure recruitment would be as complete as possible and not compromised by a failure to recruit patients who relapse early. Centres were given leave to invite patients diagnosed over the previous year provided they could be certain that no potentially eligible patients had died and could therefore not be invited to participate.

### Data collection

All patients giving written informed consent to the study are asked to complete a short family history and epidemiology questionnaire (additional files 1 and 2). Clinical data are obtained from the patient medical records by the Clinical Trials Practitioner (CTP) at each recruiting centre and entered onto a standard clinical trials data form (additional file 3). A data form is completed by the CTP at diagnosis, 6 months, 12 months and then annually from the date of definitive diagnosis. These forms capture information regarding patient characteristics, disease presentation, diagnosis and treatment, risk reducing surgery, new primary tumours, recurrence and survival (additional files 3, 4, 5). Annual follow up is continued until death, loss to follow up or until the end of the active phase of the study (January 2010).

Copies of all diagnostic breast imaging reports, cytology and pathology reports and all surgical records are collected to ensure accurate and consistent recording of tumour characteristics and TNM stage. Tumour pathology blocks are being collected and will be systematically reviewed by a panel of study pathologists on completion of recruitment to the study. Diagnostic mammograms are being collected and digitised.

### Data checking

Study files are randomly selected for periodic checking against computerised data by the study Principle Investigator (D Eccles) and co-investigator (P Simmonds) to ensure consistent data entry. The data files for recruits who die during the period of follow up are systematically checked for accuracy and consistency of data entry.

### Biological sample banking

A blood sample (30 mls) is collected from each recruit. Whole blood (for DNA) and serum are stored. Peripheral Blood Lymphocytes (PBL) are stored of cases where sample quantity and transit time permit (currently 75% of cases). Tumour tissue from paraffin blocks is collected centrally for formal pathology review and assembly of tissue micro arrays.

### Genetic risk estimation

A family history questionnaire is completed by each recruit. Reported diagnoses in the family are accepted as stated, no attempt is made to verify these diagnoses since ethical permission was not granted for this. The family history is used to rank genetic risk for each individual using the commercially available software Cyrillic^® ^which implements the BRCAPRO carrier probability algorithm since this is widely used in genetics services in the UK in particular[24]. This software does not predict with a high degree of accuracy the likelihood of BRCA1 or BRCA2 carrier status of an individual but uses family history of breast and ovarian cancer and of unaffected relatives to derive a "heterozygote risk" (ranging from 1 to 100%) with probabilities derived from population based segregation analysis [25]. In a similar way to other commonly used approaches [26] it provides an objective indication of the probability of an individual carrying a single autosomal dominant high penetrance breast cancer gene mutation. This software was used since the objective was to classify patients according to family history using a ranking system based on number of cancers, type of cancer, age at onset and modified by number of unaffected relatives in the family history. Estimating gene carrier probabilities is not highly accurate with any currently available software [27,28]. Mutation testing of BRCA1 and BRCA2 will be carried out in this cohort in the future. BRCA1 and BRCA2 mutation status is known and reported at diagnosis for a minority of recruits presenting before age 41 years (<2%) where prior contact with the clinical genetics service has allowed predictive testing for a known familial mutation prior to diagnosis. For the purposes of this analysis, the higher the estimated heterozygote risk, the greater the likelihood that an individual carries a highly penetrant dominantly inherited gene mutation. The highest risk group will include the majority but not all of the BRCA1 and BRCA2 gene carriers in the cohort. In contrast those falling into the lowest heterozygote risk groups (<20%) are most likely to be sporadic with minimal genetic component to the risk. The study criteria were set to ensure that at least 10% of the cohort will have pathogenic BRCA1 or BRCA2 mutations.

### Power calculations

The power estimates depend critically on the prevalence of mutation carriers. The initial power analyses were based on a cohort of 2000 patients and looked at a variety of possible BRCA1 prevalence rates since we intend to analyse by each gene. In a cohort of 2000 women, if the prevalence rate of BRCA1 gene carriers is 10%, a study with 200 BRCA1 gene carriers has 97% power to detect a difference in 2 year event rate of 20% in gene carriers compared with 10% for sporadic cases. If the prevalence rate of carriers drops to 5%, the number of carriers would be 100 (estimate for BRCA2) and the power drops to 78% to detect the same difference in event rates.

Approximately 30% of the participants report some family history in first or second degree relatives of breast or ovarian cancer. Preliminary mutation testing in 120 study participants revealed 1/38 (3%) of women with no documented family history of cancer had a pathogenic BRCA1 mutation and 32/81 (39.5%) of recruits with a family history of breast (or ovarian) cancer had a pathogenic mutation – 22/81 (27%) in BRCA1 and 10/81 (12%) in BRCA2. The target for recruitment was revised to 3,000 in 2006. From our preliminary mutation testing, we expect to find 260 BRCA1 gene carriers amongst women presenting with a family history (about 30% of the cohort) + 63 BRCA1 gene carriers amongst young women with no family history. We would expect 108 BRCA2 gene carriers amongst the recruits with a family history.

We will in addition have a cohort of know gene carriers but their treatment choices may differ in light of their known genetic status. The difficulty of "confounding by indication" whereby the prognosis partly determines the choice of treatment, is potentially a significant problem for non randomised trials such as this [29]. This problem can be allowed for by careful documentation of the rationale for particular treatment choices, including prophylactic surgery where this is undertaken. This information will be requested at follow up and will allow a more informative analysis to be made.

### Planned statistical analyses

Demographic data and features of the primary tumour will be compared using the two-sample t-test or the Mann-Whitney test, as dictated by the distribution of the response variable. Treatment modalities will be compared using the chi-squared test. Follow up data will be compared using Kaplan-Meier survival curves, with the log rank test being used to compare the distribution of recurrence-free times. Analysis comparing outcomes for patients treated differently will be adjusted for patient characteristics using logistic or Cox regression.

## Discussion

In the UK at present most young women diagnosed with breast cancer have not had a genetic test. Technical challenges and cost have limited the ability to perform rapid molecular genetic testing until quite recently. Thus most newly diagnosed breast cancer cases will be treated on the basis of tumour prognostic features and without knowledge of the patient's genetic status although family history is often recorded. As technology moves on and genetic testing becomes more readily available, treatment decisions may be increasingly influenced by knowledge of the patient's genetic make-up. At present such genotype directed management strategies would be premature since the published evidence is based on inherently unreliable information from small retrospective studies (reviewed in [22, 30]). The POSH study will provide unique and important data about modern breast cancer treatment in a large group of women with unusually young onset disease. It will give important insights into the underlying influences of genetic variation on disease aetiology, host response to disease and treatment effects as well as long term outcomes for early onset breast cancer.

## Competing interests

The author(s) declare that they have no competing interests.

## Authors' contributions

DE is the Chief Investigator for the POSH study, devised the study and wrote the paper. PS is co-investigator and helped design the study and database fields and co-wrote the manuscript. SG is responsible for data collection and entry and day to day management of the study. SE provided advice on data abstraction and analysis for the study, VH carried out a pilot BRCA1 and 2 mutation analysis in the cohort to verify the power estimates. DGA is involved in the planning of the study design and data analyses and advised on presentation of the manuscript.

POSH Steering Group

Additional members of the steering group for the POSH study involved in monitoring study progress and in aspects of the study design and who contributed valuable comments during the preparation of this manuscript are Dr Paul Pharoah (genetic epidemiology), Strangeways Research Laboratories, Cambridge; Professor Louise Jones (pathology), Tumour Research Laboratory, John Vane Cancer Centre, St Bartholomews Hospital, London; Professor Sunil Lakhani (pathology), University of Brisbane, Australia; Dr Ros Eeles, (Cancer Genetics Group), The Institute of Cancer Research & Royal Marsden NHS Foundation Trust; Professor Gareth Evans, (Cancer Genetics), St Mary's Hospital, Manchester; Professor Alastair Thompson (Surgery), Dundee University; Professor Shirley Hodgson (Cancer Genetics), St Georges Hospital, London; Mr Hisham Hammad (Surgery), Guy's Hospital, London; Dr Ruth Warren (Radiology), University of Cambridge; Professor Fiona Gilbert (Radiology), University of Aberdeen;

## Pre-publication history

The pre-publication history for this paper can be accessed here:



## Supplementary Material

Additional file 1 Family history questionnaire format. This shows the questions asked to elicit family history data for each recruit. Click here for file

Additional file 2 Lifestyle questionnaire format. This shows the questions asked to elicit epidemiological risk data for each recruit. Click here for file

Additional file 3 POSH diagnosis information. This shows the clinical trials form for collecting data about the initial patient diagnosis. Click here for file

Additional file 4 POSH surgery. This shows the clinical trials form for collecting data about the surgical management of each recruit. Click here for file

Additional file 5 POSH additional treatment information. This shows the clinical trials form for collecting data about adjuvant treatment for each recruit. Click here for file
