# Supplementary material for: Prospective study of Outcomes in Sporadic versus Hereditary breast cancer (POSH): study protocol
Source: BMC Cancer. 2007 Aug 15;7:160. doi: 10.1186/1471-2407-7-160 (PMC1995215; doi:10.1186/1471-2407-7-160)
Supplement: Additional file 1 — Family history questionnaire format. This shows the questions asked to elicit family history data for each recruit. [file 1471-2407-7-160-S1.doc]

Please complete the form, giving as much information as possible about your (blood) relatives, **including those who have not had cancer**.

All your information will be held in confidence in the Research team.

| **Relative** | **First name of relative**  **Or initials** | **Alive**  **Y/N** | **Current age (or age at death if relative no longer alive)** | **If your relatives suffered from cancer we would like to know the type of cancer (i.e. where the cancer first started), the age when it was diagnosed and the type of treatment that was given (if you know)** | | | |
| --- | --- | --- | --- | --- | --- | --- | --- |
|  |  |  |  | **Type of cancer** | **Age at diagnosis** | **Type of treatment (e.g. operation, tablets, chemotherapy, radiotherapy)** | |
| Self |  | Y |  |  |  |  |  |
|  |  |  |  |  |  |  |  |
| Your Children |  |  |  |  |  |  |  |
|  |  |  |  |  |  |  |  |
| Your sisters |  |  |  |  |  |  |  |
| (full or half, if |  |  |  |  |  |  |  |
| half state |  |  |  |  |  |  |  |
| which parent |  |  |  |  |  |  |  |
| you share) |  |  |  |  |  |  |  |
| Your brothers |  |  |  |  |  |  |  |
| (full or half, if |  |  |  |  |  |  |  |
| half sate |  |  |  |  |  |  |  |
| which parent |  |  |  |  |  |  |  |
| you share) |  |  |  |  |  |  |  |
| Your mother |  |  |  |  |  |  |  |
| Your father |  |  |  |  |  |  |  |

| Mother's |  | |  |  |  |  |  |  |
| --- | --- | --- | --- | --- | --- | --- | --- | --- |
| mother |  | |  |  |  |  |  |  |
| Mother's |  | |  |  |  |  |  |  |
| father |  | |  |  |  |  |  |  |
| Your father's | |  |  |  |  |  |  |  |
| mother | |  |  |  |  |  |  |  |
| Your father's | |  |  |  |  |  |  |  |
| father | |  |  |  |  |  |  |  |
| Your mother’s | |  |  |  |  |  |  |  |
| brothers | |  |  |  |  |  |  |  |
| Your mother’s sisters | |  |  |  |  |  |  |  |
|  | |  |  |  |  |  |  |  |
| Your father's | |  |  |  |  |  |  |  |
| brothers | |  |  |  |  |  |  |  |
| Your father’s | |  |  |  |  |  |  |  |
| sisters | |  |  |  |  |  |  |  |

| Any other relative |  |
| --- | --- |
| with cancer e.g. cousins. |  |
| Indicate side of the |  |
| family and who they |  |
| are related to |  |

**Version 3: Created on 1/12/03**
