# Supplementary material for: Prospective study of Outcomes in Sporadic versus Hereditary breast cancer (POSH): study protocol
Source: BMC Cancer. 2007 Aug 15;7:160. doi: 10.1186/1471-2407-7-160 (PMC1995215; doi:10.1186/1471-2407-7-160)
Supplement: Additional file 2 — Lifestyle questionnaire format. This shows the questions asked to elicit epidemiological risk data for each recruit. [file 1471-2407-7-160-S2.doc]

LOCAL HEADED PAPER

Version created 22.01.01

# MREC/00/6/69

**Title of study**: A prospective study of breast cancer treatment outcomes.

## QUESTIONNAIRE FOR PARTICIPANTS

**Please complete these few questions to the best of your ability. If you do not know the answer just indicate this on the form. ****circle the relevant answer*

NAME

DATE OF BIRTH

1. What age were you when your periods first started?
2. Are your periods regular?
3. *If yes*, what is the length of your cycle ie. how many days is it from the start of one period to the start of the next?
4. Have you ever taken the oral contraceptive pill? Y/N
   - If yes what age were you when you started?
   - How long in total have you taken it for?
   - At what age did you stop taking the pill?
5. Have you had a hysterectomy? Y/N
   - *If yes* at what age and for what reason?
6. Have you had your ovaries removed Y/N
   - *If yes* at what age and for what reason?
7. Have you ever taken hormone replacement therapy (HRT)? Y/N
   - *If yes*, when did you start?
   - Are you still taking HRT? Y/N
8. Have you ever smoked? Y/N
   - Are you currently smoking? Y/N
9. Have you ever been pregnant? Y/N
   - *If yes*, what was your age at first pregnancy?
   - What was your age at first full term pregnancy, if different from above?
   - How many children do you have?

Thank you very much for completing this questionnaire. If you would be prepare for our study coordinator or a member of the team to contact you at any stage in the future for further information please complete your address and telephone number below.

TELEPHONE NUMBER

ADDRESS
