# Supplementary material for: Prospective study of Outcomes in Sporadic versus Hereditary breast cancer (POSH): study protocol
Source: BMC Cancer. 2007 Aug 15;7:160. doi: 10.1186/1471-2407-7-160 (PMC1995215; doi:10.1186/1471-2407-7-160)
Supplement: Additional file 3 — POSH diagnosis information. This shows the clinical trials form for collecting data about the initial patient diagnosis. [file 1471-2407-7-160-S3.pdf]

POSH: Diagnosis Information

	Height (m):	Ethnic Origin:
Date Of Birth:	Weight (Kg):	Hospital Number:
Hospital:	Clinician:	

Section 1 - Menopausal Status

Please tick the status that applies

Menopausal Status

- ☐ Premenopausal
- ☐ Perimenopausal
- ☐ Postmenopausal
- ☐ Unknown

Section 2 - Presentation

Please tick the appropriate presentation then any sits of distant metastatic disease that apply

Presentation

- ☐ Incidental at breast reduction appointm
- ☐ Incidental at Breast Reduction Mammo
- ☐ Incidental at Mastectomy
- ☐ Incidental at reconstruction
- ☐ Routine Follow-up and imaging
- ☐ Screen detected-Mammo
- ☐ Screen detected-MRI
- ☐ Screen detected-Sonno
- ☐ Symptomatic

Sites Of Distant Metastatic Disease At Presentation

- ☐ Bone
- ☐ Bone Marrow
- ☐ Brain Meninges
- ☐ Brain Parenchyma
- ☐ Liver
- ☐ Lung Parenchyma
- ☐ Lung Pleura
- ☐ Mediastinal Lymph Nodes
- ☐ Other
- ☐ Ovary
- ☐ Pericardium
- ☐ Peritoneum
- ☐ Retroperitoneal Lymph Nodes
- ☐ Supraclavicular Lymph Nodes

If 'other', please specify:

Section 3 - Scan Results

Please write the result and location of any scans performed

Scan Results

	Result	Location of scan (Hospital)
Mammogram		
MRI		
PET		
Ultrasound		

Section 4 - Location Of Tumor

Please tick the location representing the site and side of the tumor

Site and side of tumor

- ☐ left
- ☐ left behind nipple
- ☐ Left Lower Inner Quadrant
- ☐ Left Lower Outer Quadrant
- ☐ left multi-quadrat
- ☐ Left Upper Inner Quadrant
- ☐ Left Upper Outer Quadrant
- ☐ right
- ☐ right behind nipple
- ☐ Right Lower Inner Quadrant
- ☐ Right Lower Outer Quadrant
- ☐ right multi-quadrat
- ☐ Right Upper Inner Quadrant
- ☐ Right Upper Outer Quadrant
- ☐ Unknown

Section 5 - Basis Of Diagnosis

Please tick the relevant basis of diagnosis prior to definitive surgery

Basis Of Diagnosis

- ☐ Clinical
- ☐ Cytology
- ☐ Excision
- ☐ Histology core biopsy
- ☐ Histology excision biopsy
- ☐ Pathological Examination
- ☐ Radiology

	Height (m):	Ethnic Origin:
Date Of Birth:	Weight (Kg):	Hospital Number:
Hospital:	Clinician:	

Section 6 - Required Documents

Please tick and return the ticked documents with this form

Copies of the following documents must be provided

	Enclosed	Not Done
Cytology Report	☐	☐
Imaging - Mammo Report	☐	☐
Imaging - MRI Report	☐	☐
Imaging - Sonno Report	☐	☐
Initial Clinical Assessment	☐	☐
Pathology - Core Biopsy Report	☐	☐
Pathology - Definitive Surgery Report	☐	☐
Pathology - Excision Biopsy Report	☐	☐
