# Supplementary material for: Prospective study of Outcomes in Sporadic versus Hereditary breast cancer (POSH): study protocol
Source: BMC Cancer. 2007 Aug 15;7:160. doi: 10.1186/1471-2407-7-160 (PMC1995215; doi:10.1186/1471-2407-7-160)
Supplement: Additional file 4 — POSH surgery. This shows the clinical trials form for collecting data about the surgical management of each recruit. [file 1471-2407-7-160-S4.pdf]

POSH: Surgery

	Height (m):	Ethnic Origin:
Date Of Birth:	Weight (Kg):	Hospital Number:
Hospital:	Clinician:	

Section 1 - Date Of Definitive Surgery

Please enter the date that the definitive surgery took place

Date of definitive surgery: __ / __ / ____

Section 2 - Definitive Surgical Treatment Of Primary Tumor

Please tick where appropriate

Surgical Treatment Of Primary Tumor

- ☐ Excision biopsy
- ☐ Nodes only
- ☐ None
- ☐ Radical mastectomy
- ☐ Segmentectomy/quadrantectomy
- ☐ Simple mastectomy
- ☐ Subcutaneous mastectomy
- ☐ Wide local excision

Section 3 - Lymph Node Procedure

Please tick the appropriate Lymph node procedure(s)

Lymph Node Procedure

- ☐ Axillary clearance
- ☐ Axillary sample
- ☐ IMC node biopsy
- ☐ lymph node disection
- ☐ SCF lymph node biopsy
- ☐ Sentinel node biopsy

Section 4 - Breast Reconstruction

If breast reconstruction has been performed, please provide the date of surgery

Date of breast reconstruction: __ / __ / ____
