# Supplementary material for: Prospective study of Outcomes in Sporadic versus Hereditary breast cancer (POSH): study protocol
Source: BMC Cancer. 2007 Aug 15;7:160. doi: 10.1186/1471-2407-7-160 (PMC1995215; doi:10.1186/1471-2407-7-160)
Supplement: Additional file 5 — POSH additional treatment information. This shows the clinical trials form for collecting data about adjuvant treatment for each recruit. [file 1471-2407-7-160-S5.pdf]

POSH: Additional Treatment For Patients With Early Stage Disease

Date Of Birth:
Hospital:
Height (m):
Weight (Kg):
Clinician:
Ethnic Origin:
Hospital Number:

NOTE: If the patient has metastatic disease then please complete the metastatic disease form

Section 1 - Chemotherapy

Tick the type of treatment administered

Treatment Type

☐ None ☐ Primary (neoadjuvant) ☐ Adjuvant

If chemotherapy was administered please complete the following:

Chemotherapy Details

Date start: __ / __ / ____ (Date commenced first cycle)

Date finish: __ / __ / ____ (Date commenced last cycle)

Number of cycles of chemotherapy: ____

☐ AC	☐ AC + Docetaxel
☐ AC + Docetaxel + Zoledronic acid	☐ AC + Docetaxel + Trastuzumab
☐ ACMF	☐ AC + Paclitaxel
☐ AC + Paclitaxel + Trastuzumab	☐ CAF
☐ capecitabine	☐ Capecitabine + Docetaxel
☐ Capcitabine + Trastuzumab	☐ capcitabine + Lapitanib
☐ Capcitabine + Zoledronic acid	☐ carboplatin
☐ Carboplatin + Docetaxel	☐ Carboplatin + Docetaxel & Trastuzumab
☐ carboplatin + 5FU	☐ Carboplatin + Gemcitabine
☐ Carboplatin + Vinorelbine + Mitomycin C	☐ CMF
☐ CMF + Doxorubicin	☐ CMF + Pacitaxel/Gemicitabine
☐ Docetaxel	☐ Docetaxel + Bevacizumab
☐ Docetaxel + Cisplatin	☐ Docetaxol + Gemcitabine
☐ Docetaxel + Trastuzmab	☐ Doxorubicin
☐ Doxorubicin + Cyclophosphamide	☐ Doxorubicin + Cylophosphamide+Docetaxel
☐ Doxorubicin + Cyclophosphamide+Paclitaxel	☐ Doxorubicin + Docetaxel
☐ EC	☐ E Carbo
☐ E Carbo F	☐ EC + Docetaxel
☐ EC + Docetaxel + Cisplatin	☐ EC + Docetaxel + Trastuzumab
☐ EC + Paclitaxel	☐ EC + Paclitaxel/Gemcitabine
☐ ECMF	☐ Epirubicin
☐ Epirubicin + capecitabine	☐ Epirubicin + Docetaxel
☐ Epirubicin + Paclitaxel	☐ Gemcitabine
☐ FEC	☐ FEC + AC
☐ FEC + CMF	☐ FEC + Docetaxel
☐ MMM	☐ Paclitaxel
☐ Paclitaxel + Gemcitabine	☐ Paclitaxel + Trastuzmab
☐ Vinorelbine	☐ Vinorelbine + Capecitabine
☐ Vinorelbine + carboplatin	☐ Vinorelbine + Epirubicin
☐ Vinorelbine + 5-Fluorouracil	☐ Vinorelbine + Tratuzmab
☐ Vinorelbine + Zoledronic acid	

☐ Other (please specify) ____

If there were any dose reductions, please fill in this section

Chemotherapy Dose Reductions

Were there any chemotherapy dose reductions? ☐ Yes ☐ No

If yes, please list below:

Cycle Number	Percentage dose reduction (as percentage of original dose)
☐	☐
☐	☐
☐	☐

	Height (m):	Ethnic Origin:
Date Of Birth:	Weight (Kg):	Hospital Number:
Hospital:	Clinician:	

NOTE: If the patient has metastatic disease then please complete the metastatic disease form

If there were any dose delays, please fill in this section

Chemotherapy Dose Delays

Were there any chemotherapy dose delays? ☐ Yes ☐ No

If yes, please list below:

Cycle Number	Number of days that treatment was delayed for
☐	☐
☐	☐
☐	☐

Date Of Birth:

Hospital:

Height (m):

Weight (Kg):

Clinician:

Ethnic Origin:

Hospital Number:

NOTE: If the patient has metastatic disease then please complete the metastatic disease form

Section 2 - Antioestrogen Therapy

If antioestrogen was administered please complete the following:

Treatment Type

☐ None ☐ Primary (neoadjuvant) ☐ Adjuvant

Date start: __ / __ / ____

Planned duration (years): ______ ☐ Is the treatment ongoing?

☐ Tamoxifen
☐ Anastrazole
☐ Letrozole
☐ Exemestane
☐ Faslodex
☐ Formestone
☐ Toremefine
☐ Megestrolacetate

Section 3 - Ovarian Suppresion

Treatment Type

☐ LHRH agonist Date Start: __ / __ / ____ Planned duration (years): ____

☐ Oophorectomy Date: __ / __ / ____

☐ Ovarian irradiation Date Start: __ / __ / ____ Planned duration (years): ____

If oophorectomy was performed, please indicate whether this was:

☐ Adjuvant treatment
☐ Prophylactic
☐ Therapeutic

Section 4 - Trastuzumab

Treatment Type

☐ None ☐ Primary (neoadjuvant) ☐ Adjuvant

Date start: __ / __ / ____

Date finish: __ / __ / ____

Schedule: ☐ weekly
☐ 3 weekly

	Height (m):	Ethnic Origin:
Date Of Birth:	Weight (Kg):	Hospital Number:
Hospital:	Clinician:	

NOTE: If the patient has metastatic disease then please complete the metastatic disease form

Section 5 - Bisphosphonates

Treatment Type

☐ None ☐ Primary (neoadjuvant) ☐ Adjuvant

Date start: __ / __ / ____

Date finish: __ / __ / ____

- ☐ Pamidronate
- ☐ Zoledronate
- ☐ Clodronate
- ☐ IBANDRONATE
- ☐ Alendronic acid
- ☐ Zoledronic acid
- ☐ Risedronate
- ☐ Calcitonin
- ☐ Other (please specify) ____

Section 6 - Radiotherapy

Treatment Type

☐ None ☐ Primary (neoadjuvant) ☐ Adjuvant

Date start: __ / __ / ____

Date finish: __ / __ / ____

Radiotherapy Fields

- ☐ Intact breast
- ☐ Intact breast + boost
- ☐ Chest wall
- ☐ Axilla
- ☐ SCF
- ☐ IMC
- ☐ Bone
- ☐ Supraclavicular Lymph Nodes
- ☐ Mediastinal Lymph Nodes
- ☐ Whole Brain
- ☐ Reconstruction
- ☐ Other
- ☐ Other (please specify) ____

Total dose: __________ Gray (Gy)

Total number of fractions: __________

Boost dose: __________ Gray (Gy)

Cutaneous reaction to Radiotherapy

- ☐ Mild
- ☐ Moderate
- ☐ Severe
- ☐ Unknown

	Height (m):	Ethnic Origin:
Date Of Birth:	Weight (Kg):	Hospital Number:
Hospital:	Clinician:	

NOTE: If the patient has metastatic disease then please complete the metastatic disease form

Section 7 - Treatment Trials	Please specify if involved in a treatment trial
